# Complete genome sequence of *Tissierella* sp. strain MB52-C2 isolated from a soil sample from Wuyi Mountain

**DOI:** 10.1128/mra.00596-26

**Published:** 2026-09-17

**Authors:** Yan Ming, Yin Li, Yina Ma, Jingjing Zhao, Guowen Dong, Wanling Qiu, Chao-Jen Shih, Yen-Chi Wu, Chih-Hung Wu, Shu-Jung Lai, Wangchung Xiao, Yanna Zhao, Sheng-Chung Chen

**Affiliations:** 1 College of Resources and Environment, Fujian Agriculture and Forestry University 12449, Fuzhou, Fujian, People’s Republic of China; 2 School of Resources and Chemical Engineering, Sanming University 66283, Sanming City, Fujian, People’s Republic of China; 3 Fujian Provincial Key Laboratory of Resources and Environmental Monitoring and Sustainable Management and Utilization, Sanming University 66283, Sanming, Fujian, People’s Republic of China; 4 College of Environment and Safety Engineering, Fuzhou University 12423, Fuzhou, Fujian, People’s Republic of China; 5 Bioresource Collection and Research Center, Food Industry Research and Development Institute 63418, Hsinchu, Taiwan, Republic of China; 6 Graduate Institute of Biomedical Sciences, China Medical University 38019, Taichung City, Taiwan, Republic of China; 7 Research Center for Cancer Biology, China Medical University 38019, Taichung City, Taiwan, Republic of China; University of Wisconsin-Madison, Madison, Wisconsin, USA

**Keywords:** *Tissierella*, soil microbiology, Wuyi Mountain

## Abstract

Here, we report the complete genome sequence of *Tissierella* sp. strain MB52-C2 (= BCRC 81439) isolated from Wuyi Mountain soil, the source region of Wuyi rock tea. The genome is 3,892,263 bp with 30.9% G + C content and supports species delineation and genomic analyses of taxonomic position and genomic features.

## ANNOUNCEMENT

Strain MB52-C2 was isolated from a tea tree’s soil sample collected from Wuyi Mountain (27.6417 °N, 117.9700 °E), Fujian, China, on 4 April 2021. The soil sample was inoculated into the anaerobic modified DSM 924 medium (1) and incubated at room temperature (~25°C) for 2 weeks. The enrichment culture was serially diluted in anoxic medium, mixed with molten agar, and rolled horizontally to form a thin agar layer, a procedure known as the roll-tube technique (2). Individual colonies were picked and repeatedly purified using the same procedure at 25°C, and then identified by 16S rRNA gene clone sequencing using the primers 8F and 1492RU (3). The purity of bacteria was confirmed by morphological observation, 16S rRNA gene analysis, and genome sequencing. The genome of strain MB52-C2 was selected to sequence for the species delineation and genomic analysis.

The strain MB52-C2 has been deposited in the Bioresource Collection and Research Center, Taiwan, as strain BCRC 81439. It was grown in the modified DSM 924 medium and incubated at 30°C. The genomic DNA of strain MB52-C2 was extracted using the NucleoBond HMW DNA Kit (Macherey-Nagel, Germany) according to the manufacturer’s instructions. The genome was sequenced at Sangon Biotech (Shanghai) Co., Ltd. using the DNBSEQ-T7 platform (MGI Tech Co., Ltd.) and MinION sequencer (Oxford Nanopore Technology).

For the DNBSEQ-T7 platform, sheared genomic DNA fragments of approximately 300 bp were utilized to prepare a 150 bp paired-end DNA library. The library preparation was performed using Hieff NGS MaxUp II DNA Library Prep Kit for Illumina (Yeasen Biotechnology [Shanghai] Co., Ltd.). The constructed library was sequenced using MGISEQ-2000RS High-Throughput Sequencing Set (PE150 format), and 8,187,330 reads were generated (Table 1) and trimmed using Trimmomatic v0.36 (4).

**TABLE 1:** Genome characteristics of *Tissierella* sp. MB52-C2

Characteristic	Value
Sequencing and assembly features	
No. of MGI-DNBSEQ-T7 total reads	81,873,300
No. of Nanopore total reads	274,977
Total no. of MGI-DNBSEQ-T7 bases	1,228,099,500 bp 6,381 bp
Total no. of Nanopore bases	1,039,832,714 bp
Genome coverage (MGI; Nanopore)	316×; 267×
Genome completeness (%); contamination (%)	98.99; 2.02
Genome features	
Chromosome size (GC content)	3,892,263 bp (30.9%)
Total no. of genes	3,888
No. of coding sequences	3,770
No. of pseudogenes	36
No. of rRNAs	18
No. of tRNAs	60
No. of noncoding RNAs	4

For MinION sequencer, 1 μg of genomic DNA was size-selected using a BluePippin system (Sage Science) with a lower cutoff of 10 kb. The sequencing library was prepared using the SQK-LSK109 Ligation Sequencing Kit and sequenced on an R9.4.1 flow cell. A total of 274,977 reads were obtained (Table 1), followed by trimming using Porechop v0.2.4 (5) and filtering using NanoFilt v2.8.0 (6). Long reads were assembled using Canu v2.2 (7) and circularized by removing one copy of the terminal overlap identified from the Canu-generated GFA output. The assembly was then polished with DNBSEQ-T7 short reads using Pilon v1.18 (8) without genome rotation. The average short- and long-read coverages were 316× and 267×, respectively. Gene predictions and annotations were performed using National Center for Biotechnology Information (NCBI) Prokaryotic Genome Annotation Pipeline (PGAP) v6.5 (9).

**Fig 1:**
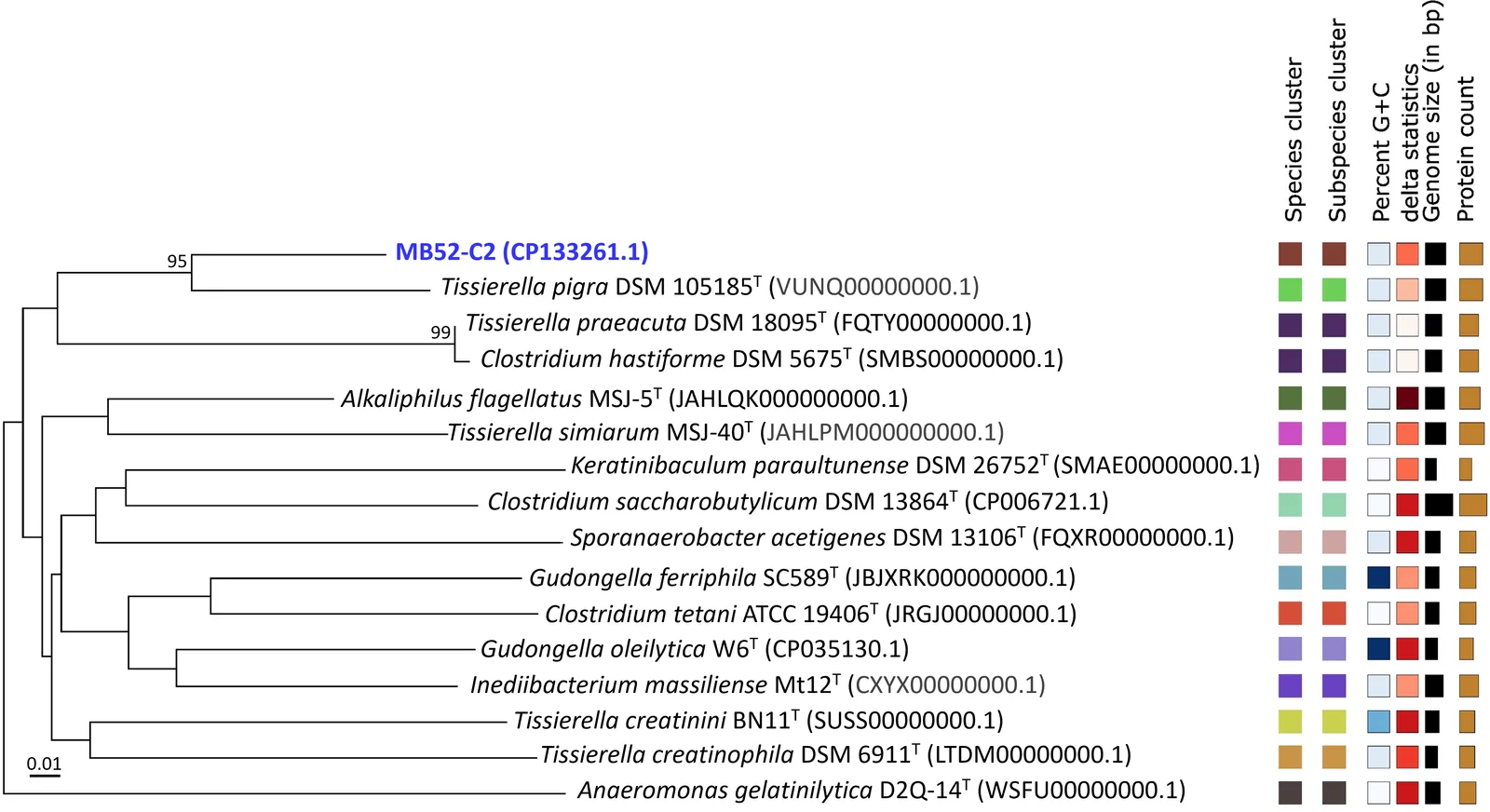
Whole genome-based phylogenetic tree of strain MB52-C2 and related type strains generated using the Type (Strain) Genome Server. The tree was inferred with FastME 2.1.6.1 (14) from Genome BLAST Distance Phylogeny distances calculated from genome sequences. Branch lengths were scaled according to GBDP distance formula d5. Numbers at the nodes indicate GBDP pseudo-bootstrap support values greater than 60% based on 100 replications. The accession number for each reference species is shown in parentheses. The colored squares indicate species clusters, subspecies clusters, genomic G + C contents, δ statistics, genome sizes, and protein counts.

The assembly generated a single large contig of 3,892,263 bp with 30.9% guanine + cystosine (G + C) content. CheckM v1.2.3 (10) estimated the genome to be 98.99% complete with 2.02% contamination (Table 1). Whole genome-based taxonomic analysis using the Type (Strain) Genome Server (TYGS) (11) placed strain MB52-C2 as a sister lineage to *Tissierella pigra* DSM 105185ᵀ with 95% GBDP pseudo-bootstrap support and assigned the two strains to separate species (Fig. 1). Furthermore, the digital DNA–DNA hybridization (dDDH) (12) and average nucleotide identity (ANI) (13) values between strain MB52-C2 and *T. pigra* DSM 105185ᵀ were 35.8 and 88.18%, respectively, below the corresponding species-delineation thresholds of 70 and 95–96%. Default parameters were used for all bioinformatics analyses.

## Data Availability

The genome sequence of strain MB52-C2 has been deposited in GenBank under accession number CP133261. The version of the genome described in this paper is the first version. The BioProject and BioSample accession numbers are PRJNA1006857 and SAMN37052310, respectively. MinION and DNBSEQ-T7 raw reads were deposited in the Sequence Read Archive (SRA) under accession numbers SRR27219901 and SRR27219902, respectively.
